# A Comparative Analysis of Mouse Imprinted and Random X-Chromosome Inactivation

**DOI:** 10.3390/epigenomes8010008

**Published:** 2024-02-10

**Authors:** Rebecca M. Malcore, Sundeep Kalantry

**Affiliations:** Department of Human Genetics, University of Michigan Medical School, Ann Arbor, MI 48105, USA

**Keywords:** imprinted X-inactivation, random X-inactivation, Xist RNA induction, X chromosome gene silencing

## Abstract

The mammalian sexes are distinguished by the X and Y chromosomes. Whereas males harbor one X and one Y chromosome, females harbor two X chromosomes. To equalize X-linked gene expression between the sexes, therian mammals have evolved X-chromosome inactivation as a dosage compensation mechanism. During X-inactivation, most genes on one of the two X chromosomes in females are transcriptionally silenced, thus equalizing X-linked gene expression between the sexes. Two forms of X-inactivation characterize eutherian mammals, imprinted and random. Imprinted X-inactivation is defined by the exclusive inactivation of the paternal X chromosome in all cells, whereas random X-inactivation results in the silencing of genes on either the paternal or maternal X chromosome in individual cells. Both forms of X-inactivation have been studied intensively in the mouse model system, which undergoes both imprinted and random X-inactivation early in embryonic development. Stable imprinted and random X-inactivation requires the induction of the Xist long non-coding RNA. Following its induction, Xist RNA recruits proteins and complexes that silence genes on the inactive-X. In this review, we present a current understanding of the mechanisms of Xist RNA induction, and, separately, the establishment and maintenance of gene silencing on the inactive-X by Xist RNA during imprinted and random X-inactivation.

## 1. Introduction

The therian mammalian sex chromosomes differ in their gene contents; whereas the X chromosome is gene-rich, the Y chromosome is gene-poor [1,2]. The unequal gene contents on the X and Y chromosomes are hypothesized to reflect their evolution. The sex chromosomes are thought to have originated from a homologous pair of autosomes [3,4,5,6,7], and the proto-Y emerged when one autosome accumulated genes favoring male sexual differentiation [1,3,5,7]. To maintain the linked segregation of male sexual differentiation genes, the proto-Y chromosome is thought to have undergone a series of crossover-suppressing inversions to form the Y chromosome [7]. Suppressed crossing over is believed to have contributed to the degradation of genes on the Y chromosome [1,3,5,8]. 

Gene loss on the Y chromosome created an imbalance in X–Y gene dosage relative to the autosomes and between XX females and XY males. To compensate for gene loss on the Y chromosome, homologous X-linked genes became upregulated in males [3,9,10]. This upregulation also occurred from both X chromosomes in females, resulting in excessive X-linked gene expression in females relative to males [3,9,10]. To rectify the overexpression of X-linked genes in females, therian mammals are believed to have evolved X-chromosome inactivation as a dosage compensation mechanism [3,11]. 

During X-inactivation, most genes on one of the two X chromosomes in females are transcriptionally silenced, thereby equalizing X-linked gene expression between females and males [11]. Two forms of X-inactivation characterize therian mammals. Metatherian mammals (marsupials) exclusively inactivate the paternal X chromosome (Xp) in a process termed imprinted X-inactivation [12,13]. Eutherian mammals (‘placentals’) undergo imprinted as well as random X-inactivation, which results in the silencing of genes on either the Xp or the Xm (maternal-X) in individual cells [11,14]. Whereas some eutherian species, e.g., mice and voles, exhibit both imprinted and random X-inactivation, others appear to only undergo random X-inactivation, e.g., humans and rabbits [14,15,16,17,18]. 

Both imprinted and random X-inactivation are established early in embryogenesis and have been studied extensively in the mouse model system. Shortly after the zygote stage, all cells in the early mouse embryo initiate imprinted X-inactivation, resulting in the silencing of genes exclusively on the Xp [18]. Imprinted X-inactivation of the Xp is subsequently maintained in the extra-embryonic trophectodermal (placental) and primitive endodermal (yolk-sac) lineages [18,19]. In the pluripotent epiblast cells of the late blastocyst (~128-cell stage), the Xp becomes reactivated [20]. As the pluripotent epiblast differentiates, each cell individually inactivates either the Xp or Xm and thus undergoes random X-inactivation [11]. Replicated copies of the randomly inactivated X chromosome are then stably maintained as inactive in most cells as the epiblast differentiates into the somatic tissues of the developing embryo. That two X chromosomes become transcriptionally divergent and that their transcriptional states are stably maintained across many cell division cycles make X-inactivation a paradigm of non-sequence-dependent, or epigenetic, transcriptional regulation. 

Stable imprinted and random X-inactivation in eutherian mammals requires the Xist long non-coding RNA (lncRNA). Xist RNA is upregulated from the future inactive-X [21] and functions in *cis* to silence genes [22,23,24]. X-inactivation can be separated into at least three phases: initiation, establishment, and maintenance. Initiation is defined as the induction of Xist RNA from the future inactive-X. Once induced, Xist RNA ‘coats’ the inactive-X and directly or indirectly recruits proteins and complexes to the inactive-X. In the establishment phase, the proteins and complexes recruited by Xist RNA silence gene expression on the future inactive-X. During the maintenance phase, the silenced state of the inactive-X is stably propagated across cell divisions in descendant cells. Discussed below is our current understanding of the mechanisms regulating Xist RNA induction and, separately, establishment and maintenance of gene silencing on the inactive X chromosome during imprinted as well as random X-inactivation. 

## 2. Regulation of *Xist* in Imprinted X-Inactivation

In imprinted X-inactivation, Xist RNA is expressed exclusively from the Xp [18]. In principle, Xist RNA expression from the Xp but not the Xm could be due to a chromatin-based mark on the Xm or the Xp. The transmission of an Xm-specific epigenetic imprint from the oocyte may prevent *Xist* expression from and inactivation of the Xm in the embryo. Conversely, the inheritance of an Xp-specific imprint from the sperm might facilitate Xist RNA expression from and inactivation of the Xp in the early female embryo. One model of the Xp imprint posits that the silencing of the sex chromosomes during meiosis in males, termed meiotic sex chromosome inactivation (MSCI), is the basis for the selective inactivation of the Xp in the early embryo [25,26]. MSCI silences genes on the X and Y sex chromosomes, which together form a heterochromatic ‘sex body’ during the pachytene stage of meiotic prophase I during spermatogenesis [27,28]. However, much work has shown that the Xp is active prior to the initiation of imprinted X-inactivation in the early embryo [29,30,31], suggesting a lack of inherited silencing of the Xp in zygotes and preimplantation embryos. Any impact of MSCI on imprinted X-inactivation must thus manifest only after the Xp becomes transcriptionally active in the zygote.

Much work has also explored whether and how the Xm carries the chromatin-based imprint in imprinted X-inactivation. Early evidence for an Xm epigenetic imprint arose from studies examining mouse embryos with supernumerary X chromosomes (e.g., XmXmY, XmXmXp). Preimplantation-stage embryos with two Xm’s resist *Xist* induction and maintain both Xm’s in a transcriptionally active state [32,33,34,35]. In contrast, individual cells in early embryos harboring two Xp’s initially express Xist RNA from both X chromosomes but later display *Xist* expression from only one of the two Xp’s [36]. These studies indicated that whereas the Xm effectively repels *Xist* induction in the early embryo, the Xp is more epigenetically labile and can both induce and repress *Xist* expression. The Xm is thus believed to carry a stringent germline imprint that prevents its inactivation during imprinted X-inactivation in the early embryo. 

The *Xist* antisense lncRNA, Tsix, has been of particular interest to understand how inactivation of the Xm is forestalled in the early mouse embryo. Whereas *Xist* is expressed from the inactive-X, *Tsix* expression marks the active-X [37,38,39]. *Xist* is thus induced from the paternal-X, and *Tsix* is expressed from the maternal-X in imprinted X-inactivation [37,39,40]. Embryos that inherit a *Tsix* mutation on the Xm ectopically express *Xist* from both the Xm (Xm-*Xist*) and the Xp (Xp-*Xist*) in cells that would otherwise exclusively express Xp-*Xist* [39,40], suggesting that *Tsix* is a *cis* repressor of Xm-*Xist*. The maternal germline imprint may thus prevent Xm-*Xist* expression in the early embryo by inducing *Tsix* expression [40]. However, tests to determine the temporal requirement of *Tsix* in repressing *Xist* during embryonic development revealed that *Tsix* is required to repress *Xist* on the Xm beginning only at the late blastocyst stage [41]. *Tsix* is thus dispensable to repress Xm-*Xist* at the onset of imprinted X-inactivation during the post-zygotic early mouse embryonic stages [41]. A *Tsix*-independent regulatory mechanism thus represses Xm-*Xist* in the zygote and preimplantation embryonic stages. 

**Figure 1:**
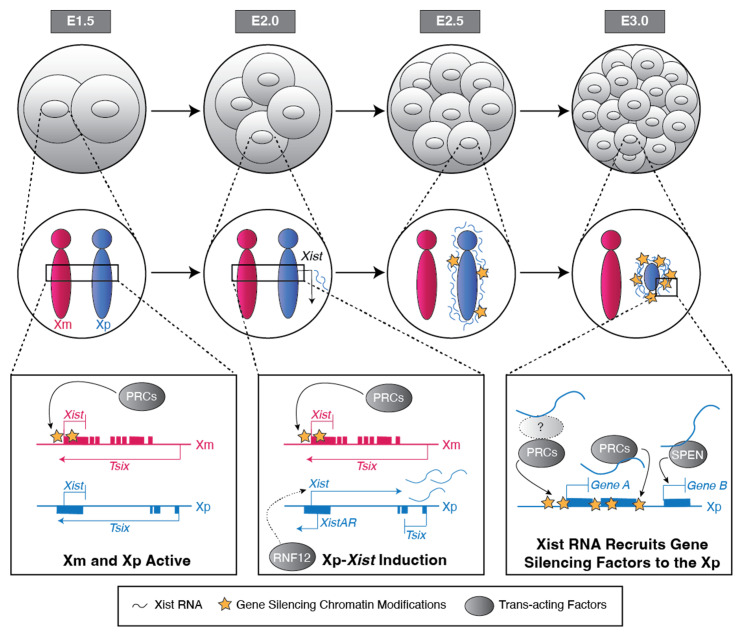
Induction of Xist RNA and establishment of gene silencing on inactive-X during imprinted X-chromosome inactivation in the early mouse embryo. Top: Diagram of early 2-, 4-, 8-, or 16-cell female mouse embryos. E, embryonic day. Middle: Depiction of the expression status of the two X chromosomes in each cell. Xm, maternal-X; Xp, paternal-X. Bottom: Schematic of the *Xist* locus or X-linked genes subject to silencing at different stages of X-inactivation. The Xm and Xp are both active prior to the initiation of imprinted X-inactivation in the early embryo. Between the 2- and 4-cell stages, the XistAR lncRNA and RNF12 protein promote Xp-*Xist* expression, whereas the Xm-*Xist* locus is silenced by PRC2-catalyzed H3K27me3 and PRC1-catalyzed H2AK119ub1. Following induction, Xp-Xist RNA directly or indirectly recruits gene silencing proteins and complexes, including PRC1, PRC2, SPEN, and SMCHD1. These proteins and complexes establish and maintain gene silencing on the Xp.

The H3 Lysine 27 tri-methylation (H3K27me3) chromatin mark emerged as a candidate Xm germline epigenetic imprint upon the discovery that H3K27me3 is enriched in the Xm-*Xist* locus in mouse oocytes and early embryos [42,43]. Xp-*Xist*, by contrast, is devoid of H3K27me3 enrichment in the sperm and early embryos (Figure 1) [42,43]. H3K27me3 is catalyzed by the Polycomb Repressive Complex 2 (PRC2) [44,45,46,47]. Upon depletion of PRC2 activity in oocytes, Xm-*Xist* is de-repressed in the early mouse embryo [43,48]. Early embryos generated from PRC2-mutant oocytes express Xist RNA from both the Xm and Xp [43,48]. In later-stage preimplantation embryos, this biallelic Xist RNA induction is stochastically resolved into one or the other X chromosome expressing *Xist* in most cells, thus resulting in randomization of X-inactivation in cells that would normally undergo imprinted X-inactivation [43]. 

In addition to PRC2, the related PRC1 complex may also ensure the repression of Xm-*Xist*. PRC1 catalyzes the gene silencing-associated histone modification H2A lysine 119 mono-ubiquitination (H2AK119ub1) [49]. H2AK119ub1, like H3K27me3, is enriched at the Xm-*Xist* locus in mouse oocytes and zygotes, but not at the Xp-*Xist* locus in sperm or early embryos [50,51]. Like the absence of PRC2 activity, the absence of the PRC1 subunits PCGF1 and PCGF6 in the oocyte results in ectopic Xm-*Xist* induction in early embryos [51]. The enrichment dynamics of H3K27me3 and H2AK119ub1 at the Xm-*Xist* locus differ during early embryogenesis. Whereas H3K27me3 is enriched at Xm-*Xist* across preimplantation development of wild-type (WT) embryos, H2AK119ub1 becomes depleted at Xm-*Xist* from the two-cell to blastocyst embryonic stages before being enriched again [50]. An interpretation of these findings is that both oocyte-derived marks are required to repress Xm-*Xist* in the early embryo and that H3K27me3 may serve to recruit PRC1 and establish H2AK119ub1 at Xm-*Xist* during preimplantation mouse embryogenesis [50].

In contrast to mice, human zygotes do not express the core PRC2 components until zygotic genome activation at the ~eight-cell stage of embryogenesis [43,52]. Imprinted X-inactivation may thus arise in a species-specific manner due to the presence of oocyte-derived silencing complexes that target Xm-*Xist* for silencing, e.g., the PRC proteins. The reasons why the oocyte-derived PRC proteins do not also target the Xp-*Xist* locus in the early mouse embryo are unclear. The resistance of Xp-*Xist* to repression may in principle arise from either the absence of factor(s) in the sperm and early embryo that function to recruit the PRCs to Xm-*Xist* in the oocyte, or, formally, the presence of a paternally inherited chromatin mark or chromosome conformation state, e.g., due to MSCI, that forestalls PRC2 occupancy at Xp-*Xist*.

SMCHD1 (structural maintenance of chromosomes hinge domain containing protein 1) also contributes to Xm-*Xist* repression in pre-implantation embryos [53]. SMCHD1 is thought to function primarily as a chromatin remodeler and compactor [54,55]. The absence of oocyte-derived SMCHD1 results in the incompletely penetrant de-repression of Xm-*Xist* in ~75% of female and ~50% of male early embryos [53]. However, Xm-*Xist* repression is restored upon zygotic SMCHD1 expression [53]. Evidence further suggests that SMCHD1 functions downstream of PRC2 and PRC1, suggesting that SMCHD1 enforces rather than initiates Xm-*Xist* silencing [53,56]. 

The induction of Xist RNA from the Xp requires an antisense transcript at the 5′ end of *Xist* called XistAR (Figure 1) [57]. Like Xist RNA, XistAR is expressed exclusively from the Xp in early mouse embryos and in cultured extraembryonic endoderm (XEN) and trophoblast stem cells (TSCs) that stably maintain imprinted X-inactivation *ex vivo* [57]. Xp-*XistAR* mutant embryos fail to properly upregulate Xist RNA and perish early in embryogenesis [57,58].

The X-linked loci *Ftx* and *Xert* have also been tested as *cis*-regulators of Xp-Xist in imprinted X-inactivation. *Ftx* resides ~150 kb upstream of *Xist* and encodes a lncRNA [59,60,61]. *Xert* is located ~200 kb upstream of *Xist*, contains an *Xist* enhancer cluster, and transcribes a lncRNA [62]. The *Ftx* locus is dispensable for imprinted X-inactivation in pre-implantation embryos [63]. However, recent findings demonstrate that deleting two enhancer elements within the *Xert* and *Ftx* loci, respectively, abrogates Xist RNA induction in eight-cell embryos [64]. This observation suggests that coordination between these *cis*-acting loci in the X-inactivation center may be necessary to upregulate *Xist* expression during imprinted X-inactivation. 

The induction of Xp-Xist RNA also requires the X chromosome-encoded protein RNF12 (also known as RLIM; Figure 1). RNF12 is an E3 ubiquitin ligase that functions in part to ubiquitinate and mark proteins for degradation [65,66]. A female-specific function of RNF12 is supported by mouse breeding data. Whereas *Rnf12*^–^*Y* hemizygous-mutant males are viable and fertile, heterozygous *Rnf12*^−/+^ female mice that inherit an *Rnf12*-mutant allele on the Xm but not the Xp are inviable [67]. *Rnf12*^−/+^ female embryos exhibit defective Xp-*Xist* upregulation and X-linked gene silencing, which may contribute to their lethality [67]. 

RNF12 is expressed more highly in female pre-implantation embryos relative to males [67]. This elevated expression of RNF12 might underpin the induction of *Xist* selectively in females. *Rnf12*^−/−^ female embryos, though, initiate but do not maintain Xp-*Xist* expression in the absence of both oocyte-derived and zygotically expressed RNF12 [68]. Furthermore, although RNF12 is required to maintain the expression of Xp-*Xist* in the early mouse embryo, it is insufficient to induce *Xist* from the Xm. These observations suggest that (1) an epigenetic imprint, i.e., H3K27me3 or H2AK119ub1, prevents RNF12-mediatied induction of Xm-*Xist*; and/or, (2) RNF12 expression is below the threshold required to induce Xm-*Xist*. In support of the latter, overexpression of RNF12 in early mouse embryos results in *Xist* induction from both the Xm and Xp [69]. 

## 3. Mechanisms of Gene Silencing on the Inactive-X during Imprinted X-Inactivation

PRC2 and PRC1 not only deposit the H3K27me3 and H2AK119ub1 marks, respectively, to repress Xm-*Xist*, but may also contribute to gene silencing on the inactive-Xp during imprinted X-inactivation. Xist RNA either directly or indirectly recruits both PRC complexes to the inactive-X [70,71]. Soon after Xp-Xist RNA induction, the Xp becomes enriched for the core components of both PRC2 and PRC1 as well as for the H3K27me3 and H2AK119ub1 marks that the two complexes catalyze [70,71,72,73,74,75]. 

Tests to determine the contributions of oocyte-derived and zygotic PRC2 revealed that PRC2 may contribute to the establishment of X-linked gene silencing during imprinted X-inactivation. Mutant female embryos lacking both the maternal (oocyte-derived) and zygotic (mz^−/−^) PRC2 core component EED as well as embryos lacking only maternal EED (m^−/−^) equally induce *Xist* randomly from either the Xm or Xp [43]. However, *Eed* mz^−/−^ embryos exhibit a greater defect in Xp gene silencing relative to *Eed* m^−/−^ embryos [43]. These observations suggest that oocyte-derived and zygotic PRC2 together may be required to establish gene silencing on the inactive-Xp during the establishment of imprinted X-inactivation [43]. 

In addition to enforcing the silencing of Xm-*Xist*, SMCHD1 may contribute to the establishment of gene silencing on the inactive-X during imprinted X-inactivation. *Smchd1*^−/+^ mutant female embryos that lack oocyte-derived SMCHD1 ectopically express a subset of paternal X-linked genes that are normally silenced in the placenta at embryonic day (E) 14.5 [53]. Formally, either the initial absence of oocyte-derived SMCHD1 or the haploinsufficiency of SMCHD1 during embryogenesis might explain this gene silencing defect. However, relative to these *Smchd1*^−/+^ mutant embryos, female heterozygotes mutant for paternal *Smchd1* and WT for maternal *Smchd1* (*Smchd1* ^+/−^) show a milder paternal X-linked gene silencing defect in the placenta [53]. Therefore, oocyte-derived SMCHD1 appears to be required to establish or maintain silencing of a subset of Xp-linked genes. Zygotically-expressed SMCHD1 may additionally contribute to the maintenance of gene silencing of a set of X-linked genes during imprinted X-inactivation [76,77]. SMCHD1 homozygous mutant (*Smchd1*^−/−^) female post-implantation embryos exhibit de-repression of a handful of X-linked genes tested thus far in the extraembryonic ectoderm and trophoblast lineages [76,77]. However, X chromosome-wide gene expression changes in the extraembryonic tissues of *Smchd1*^−/−^ female embryos await further testing. *Smchd1*^−/−^ female embryos display defective trophoblast giant cell development and do not survive past mid-gestation, whereas *Smchd1*^−/−^ males are viable [76]. The female-specific lethality of *Smchd*^−/−^ later-stage embryos is consistent with a role for SMCHD1 in maintaining imprinted X-inactivation. 

The autosomally encoded SPEN protein (also known as MINT or SHARP) is another key regulator that establishes gene silencing on the inactive-Xp (Figure 1). SPEN interacts with histone deacetylases (HDACs) [78], which help silence genes by removing acetyl marks on histones [79]. Mouse blastocyst-stage embryos lacking both oocyte-derived and zygotic SPEN (*Spen mz*^−/−^) exhibit a pronounced defect in the establishment of gene silencing on the *Xist*-expressing Xp [80]. However, early embryos lacking oocyte-derived SPEN but expressing zygotic SPEN (*Spen*^−/+^) are not defective in Xp gene silencing [80]. This suggests that expression of zygotic SPEN is sufficient to establish gene silencing during imprinted X-inactivation. Both zygotically-mutant *Spen*^−/−^ female and male embryos perish during mid-gestation between E12.5 to E14.5 [81]. The lethality of both *Spen*^−/−^ female and male embryos at a similar and relatively late embryonic stage argues against a broad and female-specific role for SPEN in X-inactivation.

PRC2 and PRC1 are also required to maintain gene silencing on the inactive-Xp (Figure 1). When zygotic PRC2 or PRC1 are absent, defects in Xp-linked gene silencing emerge in the post-implantation embryonic stages in differentiating extraembryonic cells, which normally maintain imprinted inactivation of the Xp [82,83,84]. Female embryos lacking the PRC2 subunit EED (*Eed*^−/−^) exhibit prominent Xp-linked gene silencing defects in the extraembryonic ectoderm and to some extent in the extraembryonic endoderm of post-implantation gastrulation-stage embryos [83]. Furthermore, relative to *Eed*^−/−^ male embryos, *Eed*^−/−^ female embryos exhibit reduced development of trophoblast giant cells, possibly due to defective imprinted X-inactivation in the trophectoderm lineage [85]. Similarly, female embryos lacking the PRC1 subunit RNF2 (also known as RING1B; *Rnf2*^−/−^) exhibit de-repression of X-linked genes in the extraembryonic ectoderm, albeit to a lesser extent than *Eed*^−/−^ female embryos [83]. The de-repression of X-linked genes in the extraembryonic cells of later-stage embryos in the absence of either EED or RNF2 supports the requirements of both PRC2 and PRC1 in maintaining imprinted X-inactivation of the Xp. The presence of oocyte-derived PRC2 and PRC1 components, though, may mask a broader Xp-linked gene silencing defect in the *Eed*^−/−^ and *Rnf2*^−/−^ zygotically mutant early embryos. 

The absence of PRC2 or PRC1 results in defective silencing of both overlapping and unique sets of Xp-linked genes in the extra-embryonic tissues [82,86], suggesting both redundant and independent targeting of genes on the inactive-X by PRC2 and PRC1 during imprinted X-inactivation. Loss of both PRC2 and PRC1 together in the early embryo may reveal a synergistic role for the two complexes in silencing Xp-linked genes. 

## 4. Regulation of *Xist* in Random X-Inactivation

Random X-inactivation initiates when the pluripotent embryonic epiblast begins to differentiate, just after implantation of the mouse embryo [87,88,89]. The onset of random X-inactivation has been studied to some extent in developing mouse embryos and more extensively in differentiating mouse embryonic stem cells (ESCs). Like the pluripotent epiblast cells in the developing embryo, pluripotent female ESCs harbor two active X chromosomes [90,91,92,93]. And, like the differentiating pluripotent epiblast cells in the female embryo, differentiating female ESCs randomly inactivate one of their two X chromosomes in individual cells [93]. 

In random X-inactivation, Xist RNA is induced from either the Xm or Xp in individual cells [11]. Early models of random X-inactivation posited that cellular mechanisms ‘counted’ the number of X chromosomes and then ‘chose’ one of the two X chromosomes for silencing [92,94,95]. If a cell counts two or more X chromosomes per diploid set of autosomes, it will trigger inactivation of all X chromosomes except for one. The choice step would subsequently decide which of the X chromosome(s) to inactivate.

The counting, choice, and inactivation steps underlying random X-inactivation have long been thought to be controlled by a several-hundred kilobase region of the X chromosome termed the X-inactivation center (XIC) [93,96,97]. *Xist* maps to the XIC and the XIC is thought to encode all sequences that regulate *Xist* (Table 1) [93,96,97]. The XIC is thus thought to be both necessary and sufficient to induce X-inactivation [93,96,97]. 

The XIC-derived *Xist* antisense Tsix lncRNA was put forth as a regulator of both counting and choice by controlling *Xist* expression and thus X-inactivation [37,98]. The requirement of *Tsix* in X chromosome counting was initially tested in XY male and XO ESCs. In XY and XO cells, the counting process would prevent *Xist* induction from and X-inactivation of the single X chromosome. If *Tsix* controls counting, loss of *Tsix* is expected to trigger ectopic *Xist* expression and inactivation of the single X chromosome in the XY and XO cells. Initial studies provided conflicting results for the role of *Tsix* in counting. One study suggested that deleting a 65 kb sequence containing a ~37 kb proximal portion of the *Tsix* locus might interfere with counting, as mutant XO cells ectopically expressed *Xist* [94]. Other studies with *Tsix-*mutant (*Tsix* ^−^*Y*) male ESCs reported transient ectopic *Xist* expression in a subset of the cells, which became extinguished upon extended culture [37,99], suggesting that the counting step was largely intact in the absence of *Tsix*. To test the function of *Tsix* in choice, *Tsix* heterozygous mutant (*Tsix* ^+/−^) female ESCs were differentiated to induce *Xist*. These *Tsix* ^+/−^ ESCs exhibited biased inactivation of the *Tsix*-mutant X chromosome in all cells, consistent with a defect in choice [37,40]. That only a single X is inactivated, though, suggested that X chromosome counting remained intact in *Tsix* ^+/−^ females [37,40]. 

To reconcile the disparate roles attributed to *Tsix* in X chromosome counting and choice, a set of studies examined the temporal impact of *Tsix* loss on *Xist* induction in developing embryos and differentiating pluripotent cells [41,100,101]. If *Tsix* is required for counting, *Xist* should be ectopically expressed from the single X chromosome in *Tsix* ^−^*Y* males during the early stages of differentiation corresponding to when random X-inactivation initiates in female cells. However, *Tsix* ^−^*Y* males failed to ectopically induce *Xist* early in the differentiation of pluripotent cells [100,101]. Instead, *Tsix* ^−^*Y* males ectopically expressed Xist RNA from their sole X chromosome upon further differentiation, corresponding to the stage when X-inactivation is in the maintenance phase in females [100,101]. This result suggests that *Tsix* is not required for the counting step in X-inactivation but is required to prevent inactivation of the single active-X only upon further differentiation of the cells [100,101]. 

If *Tsix* is required for the choice step of X-inactivation, *Tsix* ^+/−^ females should only exhibit *Xist* induction from the *Tsix*-mutant X chromosome and not from the WT X chromosome during the initiation stage of random X-inactivation. However, *Tsix* ^+/−^ female early embryonic epiblast cells and cultured epiblast stem cells (EpiSCs), which capture the epiblast lineage just after the establishment of X-inactivation, were found to induce *Xist* randomly from either the WT or the *Tsix*-mutant X chromosome [100,101]. After the induction of random X-inactivation, however, differentiating *Tsix* ^+/−^ cells that initially inactivated the WT X chromosome ectopically induced *Xist* from and silenced genes on the *Tsix*-mutant X chromosome, thus inactivating both X chromosomes [100,101]. Due to deficient X-linked gene expression, these cells became subject to rapid counter-selection, resulting in a surviving population of cells that had originally inactivated the *Tsix*-mutant X chromosome [100,101]. *Tsix* is thus required to prevent inactivation of the active-X only after random X-inactivation has commenced appropriately in females [100]. These findings suggest that *Tsix* is not required for the choice step in X-inactivation.

The *Jpx* locus in the XIC has also been proposed to regulate X chromosome counting and choice. *Jpx* resides ~10 kb upstream of *Xist* on the X chromosome [102]. The *Jpx* gene lacks an open reading frame and encodes a lncRNA [102,103]. Initial studies proposed that the Jpx lncRNA functions as a *trans*-acting dosage-sensitive factor to regulate X chromosome counting and choice [102,103]. Heterozygous loss of *Jpx* (*Jpx* ^+/−^) in differentiating female ESCs led to defective *Xist* induction from either of the two X chromosomes and cellular lethality [102]. Furthermore, overexpression of an autosomally integrated *Jpx* transgene caused a modest level of ectopic *Xist* induction in differentiating male ESCs [103]. 

Follow-up investigations, however, have not recapitulated the initial observations of *Jpx* function. An independent study found that *Jpx* ^+/−^ heterozygous differentiating female ESCs were viable and could induce *Xist* [104]. These *Jpx* ^+/−^ female ESCs were also able to robustly contribute to chimeras [104]. Furthermore, independent analysis of differentiating male ESCs harboring autosomal integrations of multi-copy *Jpx* transgenes did not recapitulate the ectopic *Xist* induction by *Jpx* overexpression [105]. 

In addition to these findings, a recent study revealed that *Jpx* ^−/−^ female mice are viable and are born at near-expected Mendelian ratios [106]. Moreover, embryonic fibroblasts derived from these *Jpx* ^−/−^ female embryos exhibit *Xist* expression equivalent to that of WT female fibroblasts [106]. Collectively, the above observations indicate that *Jpx* is dispensable in regulating *Xist* and random X-inactivation.

The XIC-derived Ftx lncRNA is also a proposed positive regulator of *Xist* [59,60,61]. Differentiating *Ftx* ^+/−^ female ESCs display biased *Xist* expression from the WT X chromosome [60]. Transcription of the *Ftx* locus, not the lncRNA that it produces, is thought to be required for *Xist* regulation since inhibition of *Ftx* transcription diminishes *Xist* upregulation [60]. However, *Xist* can still be upregulated in *cis* in the absence of *Ftx* and other nearby putative X-linked *Xist* regulators [104]. Furthermore, *Ftx* ^−/−^ females are born and survive to adulthood [61]. *Xist* is downregulated in *Ftx* ^−/−^ female mice, though, and a subset of X-linked genes are de-repressed, suggesting that *Ftx* is required for robust *Xist* induction and X-inactivation [61]. The mild phenotype of *Ftx* ^−/−^ female mice suggests that other factors/sequences induce *Xist* in *cis*.

The *Xert* locus and enhancer cluster within the XIC is another proposed positive *cis*-regulator of *Xist* [62]. In female ESCs, heterozygous loss of *Xert* biases *Xist* induction, with 65–80% of cells inducing Xist RNA from the WT X chromosome [62]. Furthermore, overexpression of *Xert* in male ESCs in *cis* by CRISPR activation causes ectopic *Xist* induction [62]. *Xert* may work additively with *Ftx* to induce *Xist* in *cis*, as heterozygous deletion of *Xert* and *Ftx* in ESCs together leads to expression of Xist RNA exclusively from the WT X [62]. The additive functions of *Xert* and *Ftx in vitro* suggest that the combined loss of *Xert* and *Ftx* may result in a more pronounced developmental phenotype relative to *Ftx* ^−/−^ female mice. A definitive test of the requirement of *Xert* alone or together with *Ftx* in inducing *Xist* and random X-inactivation awaits the generation of mutant mice. 

In contrast to *Ftx* and *Xert*, the X-linked *Linx* locus is thought to negatively regulate *Xist* in *cis* [107,108]. *Linx* maps within the XIC ~150 kb downstream of the *Xist* promoter and is co-expressed with *Tsix* in the embryonic lineage before random X-inactivation [107,108]. *Linx* could, therefore, function independently or through the regulation of *Tsix* to negatively regulate *Xist*. Heterozygous deletion of the *Linx* promoter, though, does not alter *Tsix* expression [107]. In female post-implantation embryos, heterozygous deletion of the *Linx* promoter results in a modest bias (54%) in X-inactivation, favoring the mutant X chromosome [107]. More recently, the Lppnx lncRNA has been described to arise from the region corresponding to the *Linx* locus [109,110]. Deletion of the *Lppnx* promoter, which appears to be nearly identical to the *Linx* promoter, similarly results in a modest bias in X-inactivation of the mutant X in mid-gestational female embryos [109,110]. Due to the repetitive sequences in the *Linx/Lppnx* region, whether these two loci are distinct remains unclear [111]. The mild effects observed upon deletion of the *Linx/Lppnx* promoters, however, suggest that other factors contribute to *Xist* repression.

A competing model posits that physical pairing of the two X chromosomes at the onset of random X-inactivation in females underlies X chromosome counting and choice. Early studies using DNA fluorescent *in situ* hybridization in differentiating female ESCs demonstrated that loci near *Xist* on the two X chromosomes transiently co-localize at the onset of random X-inactivation [112,113,114]. These studies posited that this transient co-localization facilitates counting and choice of only one of the two X chromosomes for the induction of *Xist* and X-inactivation [112,113,114]. Several XIC loci have been nominated to modulate X-pairing, including *Tsix*, *Xite*, and *Xpr* [112,113,115]. *Xite* is a lncRNA-expressing sequence thought to enhance *Tsix* expression [116,117]. *Xpr* (X-pairing region) contains the *Xpct* (*Slc16a2*) gene, which encodes for a thyroid hormone transporter [113]. Initial work showed that the *Xpr* sequence could trigger ectopic *Xist* expression in a subset of cells when autosomally integrated [112,113]. 

Several pieces of evidence collected more recently suggest that X-pairing may not be essential for counting and choice in females. Large heterozygous deletions encompassing the putative X-pairing loci *Tsix*, *Xite*, and the *Xpr* do not abrogate *Xist* induction [104,118]. Furthermore, XX-XY heterokaryons that contain separate XX and XY nuclei in a shared cytoplasm nevertheless exhibit Xist RNA coating of the sole X chromosome in the XY nuclei at a frequency consistent with independent initiation of X-inactivation of one of the three X chromosomes in these cells [104]. This finding suggests that in the absence of pairing, the X chromosome in the XY nucleus of an XX-XY heterokaryon is capable of inducing *Xist* [104]. Separately, another set of experiments attempted to prevent pairing in female ESCs by tethering one or both X chromosomes to the nuclear lamina and found that Xist RNA was still induced from one of the two X chromosomes upon the tethering [119]. Taken together, these experiments suggest that X-pairing is not required for either the counting or choice steps of X-inactivation. 

**Figure 2:**
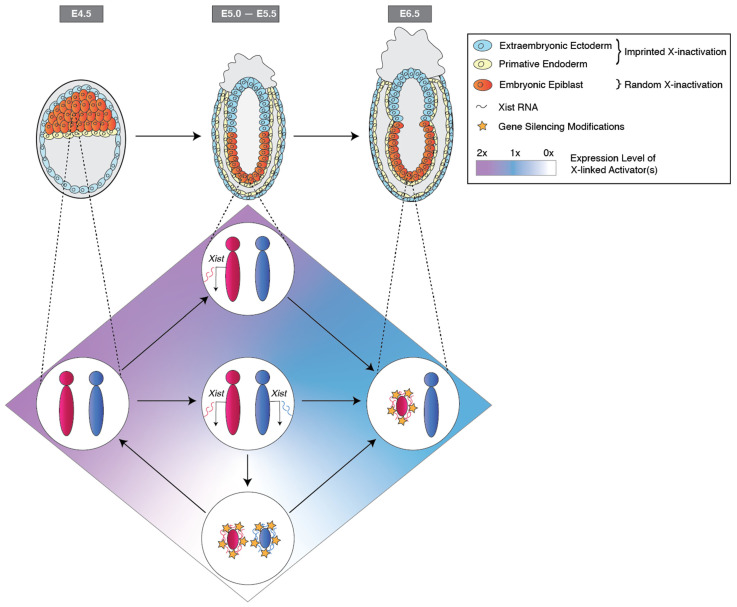
Stochastic *Xist* induction during random X-chromosome inactivation in the mouse embryo. The Xp is reactivated by embryonic day (E) ~4.5 in the female embryo. At this stage, both the Xm and Xp express activators of *Xist*. Increased expression of X-linked activators stochastically induces *Xist* on the Xm and/or Xp between E5.0 to E5.5. Individual embryonic epiblast cells transiently exhibit distinct patterns of *Xist* induction. In cases where both X chromosomes induce *Xist*, both X chromosomes may initiate silencing, which in turn results in a reduction of expression of the X-linked *Xist* activators. Reduced expression of *Xist* activators leads to a loss of *Xist* induction. This feedback loop resolves only when Xist RNA is expressed from a single X chromosome and that single X chromosome is transcriptionally silenced in female cells. Upon inactivation of a single X chromosome, the expression of X-linked activator(s) of *Xist* reaches an intermediate level, thereby preventing induction of *Xist* from the active X chromosome in female cells.

Recent time-course observations of Xist RNA expression challenge the counting and choice model of X-inactivation. These analyses have revealed transient biallelic expression of *Xist* from the two parental X chromosomes when *Xist* is first induced in the epiblast lineage of developing WT female mouse embryos (Figure 2) [87,88,120,121]. The counting and choice model predicts monoallelic induction of *Xist* from one of the two X chromosomes at the onset of random X-inactivation in female cells [121,122]. Thus, biallelic induction of *Xist* from both X chromosomes at the onset of random X-inactivation argues against the counting and choice model of random X-inactivation (Figure 2). Rather, the induction of *Xist* from both X chromosomes suggests that the activity and expression dose of one or more X-linked *trans*-acting factors suffices to induce *Xist* during the initiation of random X-inactivation [121,122,123]. Upon the stochastic and non-synchronous induction of *Xist* from both alleles, the expression of X-linked inducer(s) of *Xist* is expected to be reduced, thereby preventing robust *Xist* induction from both X chromosomes (Figure 2) [121,122]. 

*Rnf12*, which may fall within the XIC, was the first X chromosome encoded protein proposed to be a stochastic inducer of *Xist* [121]. *Rnf12* ^+/−^ female ESCs exhibit reduced *Xist* expression levels [104]. Conversely, transgenic overexpression of RNF12 in male ESCs results in ectopic *Xist* induction [105]. *Rnf12* is amongst the earliest genes to be silenced when X-inactivation is being established [29,105,121], thereby potentially preventing robust *Xist* induction from both X chromosomes. RNF12, therefore, matches the stipulations of an X-linked factor that stochastically induces *Xist* in female cells. However, the epiblast-specific loss of RNF12 in female mouse embryos does not ablate *Xist* expression or cause lethality [124]. These *Rnf12*^−/−^ epiblast cells, though, do show reduced Xist RNA expression relative to WT female epiblast cells [124]. RNF12 may thus be required for robust *Xist* induction during random X-inactivation [124]. Nevertheless, these results suggest that additional X-linked factors act in *trans* to stochastically induce *Xist*, either independently of or together with RNF12.

The X-linked KDM5C demethylase of histone H3 lysine 4 di- and trimethylation (H3K4me2/3) [125,126,127] has also been recently shown to induce *Xist* in a dose-dependent manner [128]. *Kdm5c* ^−/−^ females are inviable, whereas *Kdm5c* ^−^*Y* males are viable and fertile [128,129]. *Kdm5c* ^−/−^ female embryos and differentiating female ESCs exhibit a >80% reduction in *Xist* induction relative to WT female embryos [128]. KDM5C over-expression ectopically induces *Xist* in male ESCs, suggesting that KDM5C functions in a dose-dependent manner to induce *Xist* [128]. *Kdm5c* escapes X-inactivation and is expressed from both the active and the inactive-X chromosomes in females [130]. However, upon the establishment of X-inactivation, *Kdm5c* expression is reduced relative to when both Xs are active prior to X-inactivation [130,131]. Thus, although *Kdm5c* escapes X- inactivation, the downregulation of KDM5C expression upon the commencement of X-inactivation may forestall robust *Xist* induction and inactivation of the second X chromosome. KDM5C function and expression dynamics, therefore, are consistent with KDM5C inducing *Xist* in a dose-dependent and stochastic manner.

Like KDM5C, KDM6A is an X-inactivation escapee that has also been suggested to induce Xist RNA [130,132]. KDM6A functions as a demethylase of H3K27me3 [133,134,135]. Loss of KDM6A results in defective *Xist* induction in differentiating female ESCs [132]. *Kdm6a* homozygous mutant female (*Kdm6a*^−/−^) mice perish midway through gestation between E10.5 to E12.5, whereas *Kdm6a*^−^*Y* males are viable and fertile [136]. Whether the lethality of *Kdm6a*^−/−^ female mice is related to defective random X-inactivation awaits the characterization of *Kdm6a*^−/−^ embryos. The absence of *Rnf12*, *Kdm5c*, or *Kdm6a* individually does not appear to completely abrogate Xist RNA induction [124,128,132]. It is thus likely that multiple X-linked factors function cooperatively in a dose-dependent manner to robustly induce *Xist* in females. 

The *Kdm5c* and *Kdm6a* loci both reside far outside the XIC. That these non-XIC-encoded factors are required for *Xist* induction during random X-inactivation challenges the notion that all *Xist* regulators reside in the XIC. These observations prompt the exploration of other non-XIC X-linked loci in regulating *Xist* and random X-inactivation. 

Emerging evidence also suggests that the autosomally encoded SPEN protein induces *Xist* during random X-inactivation [137]. *Spen*^−/−^ female ESCs were initially reported to exhibit defective *Xist* induction upon differentiation [137]. However, a separate study of *Spen*^−/−^ female ESCs did not replicate this observation [138]. Since SPEN is autosomally encoded, its expression is expected to be equal between females and males. To induce *Xist* selectively in females, SPEN may need to be recruited to the *Xist* locus either directly or indirectly by X chromosome-encoded factors that are differentially expressed between females and males. 

## 5. Mechanisms of Gene Silencing on the Inactive-X during Random X-Inactivation

SPEN has a significant role in establishing X-linked gene silencing during random X-inactivation. By profiling the proteins that bind Xist RNA, several independent groups found that SPEN directly interacts with Xist RNA [78,139,140]. SPEN interacts with Xist RNA’s repeat A through an RNA-recognition motif (RRM) [78,139,141]. Xist repeat A is required for robust Xist RNA-mediated X-linked gene silencing, and the loss of SPEN results in a similar X-linked gene silencing defect relative to the loss of repeat A [142,143]. 

SPEN is recruited to the inactive X concomitantly with Xist RNA induction, suggesting that SPEN contributes to the establishment of X-linked gene silencing during random X-inactivation [80]. SPEN harbors a SPOC domain (Spen paralog and ortholog C-terminal domain) that is necessary for SPEN-mediated X-linked gene silencing [78,80,144]. The SPEN SPOC domain interacts with the SMRT/NCoR co-repressor complex, which recruits histone deacetylases (HDACs) to target loci [78,80,144,145]. By deacetylating histone tails, HDACs may contribute to X-linked gene silencing [144]. However, ablation of the SPOC domain does not completely abrogate X-linked gene silencing [146], suggesting that other domains of SPEN and/or other factors contribute to the establishment of X-linked gene silencing during random X-inactivation. The absence of any reported female-specific defects and the relatively late lethality of *Spen*^-/-^ female and male embryos (E12.5–E14.5), though, argue against an essential role of SPEN in the establishment of random X-inactivation [81]. 

PRC2 and PRC1 also contribute to X-linked gene silencing establishment during random X-inactivation. Loss of the core PRC2 subunit SUZ12 in female ESCs leads to defective establishment of silencing of a subset of X-linked genes upon differentiation [143]. Similarly, ablation of the PRC1 subunits PCGF3 and PCGF5 in differentiating female ESCs results in defective silencing of a subset of X-linked genes [146]. The loss of PCGF3 and PCGF5 also abrogates Xist RNA-mediated recruitment and accumulation of PRC1-catalyzed H2AK119ub1 and to some extent PRC2-catalyzed H3K27me3 on X-linked genes [143,147,148]. The impact of PCGF3 and PCGF5 loss on H3K27me3 accumulation has led to the proposal that PRC1 recruits PRC2 to loci on the inactive-X [143,147,148]. The order of recruitment of the two PRC complexes to the inactive-X has been the subject of much recent debate. 

Some studies have suggested that PRC2 and PRC1 directly interact with Xist RNA [149], whereas others propose that PRC2 and PRC1 are recruited through indirect interactions with Xist RNA [147,148,150]. Various approaches using crosslinking followed by mass spectrometry (MS) have not consistently shown direct interactions between the subunits of PRC2 or PRC1 with Xist RNA. One study that employed UV-crosslinking identified the PRC2 subunit EZH2 as one of more than 100 hits inferred to be direct Xist RNA interactors [140]. A different study using formaldehyde crosslinking found several subunits of PRC1, but not PRC2, to directly interact with Xist RNA [139]. However, profiling of direct protein–Xist RNA interactions using UV-crosslinking after stable isotope labelling of amino acids in culture (SILAC) for quantitative MS did not yield any subunits of PRC2 or PRC1 [78]. Xist RNA may thus recruit PRC2 and PRC1 to the inactive-X indirectly. PRC2 may be indirectly recruited to the inactive-X through a direct interaction of the PRC2 accessory subunit JARID2 with Xist RNA [151]. Similarly, the direct Xist RNA-binding protein hnRNPK may be an indirect recruiter of PRC1 to the inactive-X [139,148]. 

SMCHD1 is also thought to maintain X-linked gene silencing during random X-inactivation downstream of the PRCs and SPEN [146]. SMCHD1 has been proposed to interact directly with Xist RNA [140]. However, a study using PAR-CLIP (photoactivatable ribonucleoside-enhanced cross linking and immunoprecipitation) found that SMCHD1 does not directly interact with any RNAs, including Xist RNA [152]. Instead, evidence suggests that SMCHD1 is indirectly recruited to the inactive-X through its interaction with hnRNPK, similar to the proposed mechanism of hnRNPK recruiting PRC1 to the inactive-X [148,152]. In support of this mechanism, loss of Xist RNA repeats B and C, to which hnRNPK binds, ablates SMCHD1 accumulation on the inactive-X [152]. The recruitment of SMCHD1 downstream of PRC1 is consistent with a primary role of SMCHD1 in maintaining X-linked gene silencing [146]. 

## 6. Open Questions

Our understanding of the necessity of *cis*-acting loci within the XIC in regulating *Xist* expression during imprinted X-inactivation is limited relative to random X-inactivation (Table 1). The XistAR and Tsix lncRNAs have established roles in Xp-*Xist* induction and in the maintenance of Xm-*Xist* repression, respectively, during imprinted X-inactivation [41,57]. In contrast, other *cis*-acting loci either do not appear to have a role or await further testing as regulators of *Xist* in imprinted X-inactivation.

**Table 1:** Summary of factors regulating Xist RNA induction during imprinted vs. random X-inactivation. All studies were performed in mouse embryos or cells.

Region	Regulatory Factor	Imprinted X-Inactivation	Random X-Inactivation
**XIC**		***Xist* Expression in Mutants**	***Xist* Expression in Mutants**
	**Tsix lncRNA**	Xm-*Xist ↑↑↑* *Tsix* ^−/+^ pre-implantation embryos [41]	*Xist* ↑↑↑ from WT active-X *Tsix* ^+/−^ post-implantation embryonic epiblasts [100]
	**XistAR lncRNA**	Xp-*Xist* ↓↓↓ *XistAR* ^+/−^ pre-implantation embryos [57]	Biased *Xist* expression from WT X *XistAR* ^+/–^ EpiSCs [57]
	**Jpx lncRNA**	Awaits further testing	No reported change *Jpx* ^−/−^ mouse embryonic fibroblasts [106]
	***Ftx* locus and/or lncRNA**	No change *Ftx* ^+/−^ pre-implantation embryos [63]	Biased *Xist* expression from WT X Differentiating *Ftx* ^+/−^ ESCs [60]
	***Xert* locus and/or lncRNA**	Awaits further testing	Biased *Xist* expression from WT X *Xert* ^+/−^ promoter deletion; differentiating ESCs [62]
	***Linx/Lppnx* locus and/or lncRNA**	Awaits further testing	Biased *Xist* expression from mutant X *Linx/Lppnx* ^+/−^ promoter deletion; post-implantation embryonic epiblasts [107,109]
	**RNF12**	Xp-*Xist* ↓↓↓ *Rnf12* ^−*/+*^ and *Rnf12* ^−/−^ pre-implantation embryos [67,68]	*Xist* ↓ *Rnf12* ^−/−^ post-implantation embryonic epiblasts [124]
**Non-XIC**	**KDM5C**	No reported change *Kdm5c* ^−/−^ pre-implantation embryos [128]	*Xist* ↓↓↓ *Kdm5c* ^−/−^ post-implantation embryonic epiblasts [128]
	**KDM6A**	Awaits further testing	*Xist* ↓↓ Differentiating *Kdm6a^–/–^* ESCs [132]
**Auto-** **somal**	**SPEN**	No reported change *Spen* ^−/−^ pre-implantation embryos [80]	Variable reports [137,138]
	**PRC2/1**	Randomized *Xist* expression Pre-implantation embryos lacking oocyte-derived PRC2 or PRC1 [43,48,51]	No reported change PRC2 or PRC1 null post-implantation embryonic epiblasts [82,153]
	**SMCHD1**	Incompletely penetrant Xm-*Xist ↑* Pre-implantation embryos lacking oocyte-derived SMCHD1 [53]	No reported change *Smchd1* ^−/−^ post-implantation embryos [76]

Distinct *trans*-acting factors regulate *Xist* during imprinted vs. random X-inactivation [121,122] (Table 1). For example, PRC2 and PRC1 are required to prevent *Xist* expression from the active X (the Xm) during imprinted X-inactivation but not during random X-inactivation [67,68]. Conversely, KDM5C and SPEN appear to be required for *Xist* induction during random but not imprinted X-inactivation [80,128].

Testing the requirement of factors for *Xist* induction in both imprinted and random X-inactivation can be made more conclusive through the examination of mutant mouse embryos. Many factors implicated in the induction of random X-inactivation have been tested in stem cells but not in embryos (Table 1). Some factors, e.g., SPEN, exhibit varied results in stem cell models of random X-inactivation [103,104,137,138]; therefore, conducting tests in embryos may provide deeper insights. 

## 7. Conclusions

Our improved understanding of Xist RNA induction as well as the establishment and maintenance of X-linked gene silencing during X-inactivation derives from technological advances, which have propelled a re-examination of previous conclusions. Although the mechanisms of Xist RNA induction during imprinted and random X-inactivation are distinct, both forms of X-inactivation appear to rely largely on the same set of factors to establish and maintain X-linked gene silencing. The use of mouse embryos has provided valuable insights into the mechanisms of both imprinted and random X-inactivation, as the embryos undergo both types of inactivation. The extent to which the models derived from the mouse apply to the onset of X-inactivation in other therian mammals, including humans, remains largely unknown and will be important to elucidate in the future.

## Data Availability

Not applicable.
